# To Go Where Nature Leads: Focus on Palmitoylethanolamide and Related ALIAmides as Innovative Approach to Neuroinflammatory and Pain-Related Disease States in Honor of Doctor Francesco Della Valle

**DOI:** 10.3390/biom13111583

**Published:** 2023-10-26

**Authors:** Salvatore Cuzzocrea, Rosalia Crupi

**Affiliations:** 1 Department of Chemical, Biological, Pharmaceutical and Environmental Sciences, University of Messina, 98166 Messina, Italy; 2 Department of Veterinary Science, University of Messina, 98166 Messina, Italy

Dr. Francesco Della Valle made a significant impact in both the business and scientific domains. Reading for a degree in chemistry, with an emphasis on organic and biological disciplines, he quickly distinguished himself in the pharmaceutical industry. This distinction was largely due to the industrial ventures he passionately and adeptly led. His fervor for science resulted in him receiving three honorary degrees and co-inventing numerous pharmaceutical patents, including more than 100 in Europe, with broad recognition in major countries, and as many active patents in the United States.

A quintessential man of science, Dr. Della Valle was simultaneously a visionary entrepreneur always in search of innovation and a thorough, meticulous manager who steered his company with determination. Recognizing the importance of open innovation, he valued the synergistic relationships between academia and industry [1].

A devoted scholar of neuroscience, Dr. Della Valle collaborated with Nobel laureate Rita Levi Montalcini for many years. He deeply grasped the significance of biological breakthroughs like the nerve growth factor (NGF), drawing him closer to Professor Montalcini [2]. “Try to understand the strategy that Nature would use to protect itself from harm”, was the counsel given to him by the Nobel laureate in 1991 [3]. Inspired by this advice, Della Valle pursued the discovery and development of substances that could emulate nature’s protective strategies through the ALIA mechanism [4]. He was profoundly invested in studying neuroinflammation as the foundation for various ailments, ranging from neuropathic pain to neurological diseases [5]. His scientific journey was marked by a relentless “quest for life”, gleaning insights and motivation from nature.

Dr. Francesco Della Valle leaves behind a legacy of pharmaceutical patents, scientific publications, pharmacological innovations, and concepts of neuroinflammation and neuropathic pain. Yet, his most invaluable legacy is his passion for science, truth, dialogue, and life itself.

The research presented in this Issue underscores the importance of continuing studies on PEA and related ALIAmides, using insights from nature to tackle new and increasingly complex challenges. We would like to express a heartfelt thank you to all the authors, reviewers, and contributors who have made the publication of this Special Issue possible. Our hope is that the information shared will inspire further research and exploration.

Here is a List of contributions contained in the Special Issue.
